# Primary Cilia Exhibit Mechanosensitivity to Cyclic Tensile Strain and Lineage-Dependent Expression in Adipose-Derived Stem Cells

**DOI:** 10.1038/s41598-019-43351-y

**Published:** 2019-05-29

**Authors:** Josephine Bodle, Mehdi S. Hamouda, Shaobo Cai, Ramey B. Williams, Susan H. Bernacki, Elizabeth G. Loboa

**Affiliations:** 1 Joint Department of Biomedical Engineering, University of North Carolina at Chapel Hill and North Carolina State University, Raleigh, North Carolina 27695 USA; 2 Department of Materials Science and Engineering, North Carolina State University, Raleigh, North Carolina 27695 USA; 3 College of Engineering at University of Missouri, W1051 Thomas & Nell Lafferre Hall, Columbia, MO 65211 USA

**Keywords:** Mesenchymal stem cells, Mesenchymal stem cells, Stem-cell differentiation, Stem-cell research, Translational research

## Abstract

Non-motile primary cilia are dynamic cellular sensory structures and are expressed in adipose-derived stem cells (ASCs). We have previously shown that primary cilia are involved in chemically-induced osteogenic differentiation of human ASC (hASCs) *in vitro*. Further, we have reported that 10% cyclic tensile strain (1 Hz, 4 hours/day) enhances hASC osteogenesis. We hypothesize that primary cilia respond to cyclic tensile strain in a lineage dependent manner and that their mechanosensitivity may regulate the dynamics of signaling pathways localized to the cilium. We found that hASC morphology, cilia length and cilia conformation varied in response to culture in complete growth, osteogenic differentiation, or adipogenic differentiation medium, with the longest cilia expressed in adipogenically differentiating cells. Further, we show that cyclic tensile strain both enhances osteogenic differentiation of hASCs while it suppresses adipogenic differentiation as evidenced by upregulation of *RUNX2* gene expression and downregulation of *PPARG* and *IGF*-*1*, respectively. This study demonstrates that hASC primary cilia exhibit mechanosensitivity to cyclic tensile strain and lineage-dependent expression, which may in part regulate signaling pathways localized to the primary cilium during the differentiation process. We highlight the importance of the primary cilium structure in mechanosensing and lineage specification and surmise that this structure may be a novel target in manipulating hASC for in tissue engineering applications.

## Introduction

Human adipose-derived stem cells (hASCs) are a multipotent stem cell type that can be easily isolated from excess fat tissue. Due to their ability to differentiate along a variety of mesenchymal lineages, they are being actively explored for their potential use in a wide range of tissue engineering and regenerative medicine therapeutics^1–3^. However, many of the underlying mechanisms of hASC lineage specification have yet to be elucidated.

hASC are capable of differentiating into adipogenic, osteogenic, chondrogenic, vasculogenic and myogenic lineages^1,2,4–6^. Specific combinations of chemical, mechanical and environmental factors are known to induce hASC differentiation towards these various cell phenotypes^1,2,7–12^. Previous work from our group and others, has shown that providing physiologically relevant mechanical stimulation can enhance hASC lineage specification^4,7,12–16^. This is particularly observed when directing hASCs toward musculoskeletal cell phenotypes, which have a largely mechanical function *in vivo*. More specifically, we have shown that hASCs exhibit enhanced osteogenic differentiation via an increase in calcium accretion when cultured under 10% cyclic tensile strain and osteogenic differentiation media (ODM) as compared to hASCs cultured in ODM in static culture^7,17^. Other groups have reported an enhanced osteogenic phenotype in hASCs when cultured under fluid flow conditions^13^. Further, this mechanically controlled phenotypic development has also been observed in hASC-derived chondrogenic^14^ and myogenic lineages^4^.

The mechanisms behind mechanical sensing and signal transduction have been investigated and these studies have largely implicated the cytoskeleton as well as cell-cell and cell-matrix interactions as sensors and effectors of mechanical stimuli^18^. We propose that the primary cilium may also be another important cell structure involved in this process. Primary cilia are known to act as mechanosensors in bone and kidney epithelial cells when exposed to fluid shear^19^, and are also known to change their length in response to their mechanical environment in tissues such as tendon and cartilage^20,21^. More recently, it has been shown that primary cilia exhibit dynamic length fluctuations during adipogenic differentiation throughout the duration of *in vitro* adipogenic differentiation in human mesenchymal stem cells (hMSCs) and hASCs^22,23^.

Primary cilia are non-motile cilia, which can be observed on nearly every mammalian cell type at some point during the cell cycle. The primary cilium emanates from the basal body, formed by the mother centriole, and it is composed of nine microtubule doublets arranged in a 9 + 0 configuration, with nine concentric tubulin doublets forming the axoneme of the cilium. They are structurally distinct from motile cilia (9 + 2), which are present in the mucosal epithelial layers of tissues such as the lung and intestinal tract and have a 9 + 2 microtubule configuration with an additional central pair of microtubules. Previous work from our group has shown that hASCs express primary cilia on anywhere from 20–65% of their cell population, without serum starvation, depending on confluency^24^. Further, we have shown that the expression of cilia-associated proteins Polycystin 1 (PC1) and Intraflagellar transport protein 88 (IFT88) play an important role in the osteogenic differentiation capacity of hASCs^24^. Under chemically-induced osteogenic differentiation, siRNA knockdown of *PC1* and *IFT88* conferred a reduction in hASC osteogenic differentiation. These data implicate primary cilia as chemosensitve cell organelles.

During the process of cell lineage specification, cells change and reorganize their cytoskeleton thus changing their cell morphology, characteristic of a particular phenotype^25^. In *in vitro* model systems, physically controlling cell morphology also modulates phenotypic specification in hMSCs and ASCs^26,27^. Moreover, applying mechanical stimulation to stem cells effects changes in lineage commitment signals, often enhancing chemically induced phenotypes^9,12,13,28–30^. The primary cilium is somewhat contiguous with the microtubule cytoskeleton via the docking of the centrosome at the apical surface of the cell^31^. Thus it follows that the cilium structure itself may be sensitive to morphological changes effected by cytoskeletal reorganization in response to mechanical cues^32^. Conversely, the cilium structure is known to be a mechanosensing organelle in many tissues and in the case of stem cell differentiation, it may play a part in facilitating the mechanically-induced cytoskeletal reorganization.

Based on our previous work, we have established that primary cilia-associated proteins are involved in hASC osteogenic differentiation^24^ and that tensile strain enhances hASC osteogenesis^7,12,16^, and thus we hypothesize that the mechano-active primary cilium may be a critical structure in this process^32^. We postulate that the cilium structure is intimately involved in lineage specification processes and that it dynamically modulates and/or is modulated by chemically- and mechanically- induced hASC differentiation.

## Results

### Cyclic tensile strain enforces cellular alignment and differentially affects calcium accretion and lipid accumulation in osteogenic and adipogenic cells, respectively

**Figure 1:**
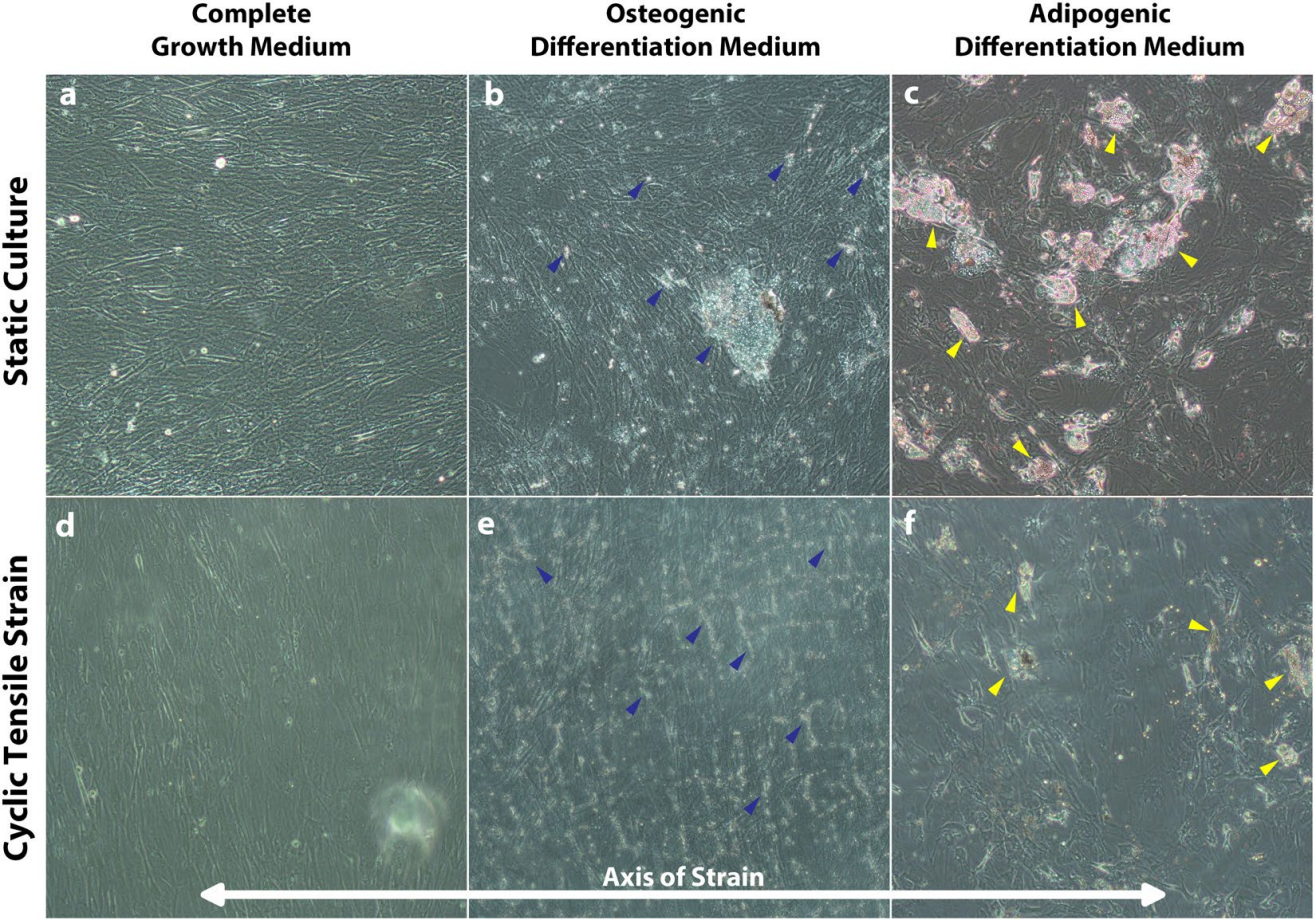
hASCs exhibit different cellular morphology in response to both chemical and mechanical stimulation. hASC cultured under complete growth medium (CGM) (**a**,**d**) osteogenic differentiation medium (ODM) (**b**,**e**) and adipogenic differentiation medium (ADM) (**c**,**f**) for 17 days either in static culture (**a–c**) or under 10% cyclic tensile strain (4 hours/day, 1 Hz). (**d–f**) Accumulation of calcium deposits (blue arrows) indicate osteogenic differentiation in both static and strained osteogenic hASC. (**b**,**e**) Lipid droplet accumulation within cells (yellow arrows) indicates adipogenesis in both static and strained adipogenic hASC. (**c**,**f**) No demarcated evidence of differentiation is observed in CGM, however all hASC exposed to strain tend to align perpendicular to the axis of strain (**d–f**).

When cultured in complete growth medium (CGM), osteogenic differentiation medium (ODM) or adipogenic differentiation medium (ADM) over the course of 17 days, hASCs begin to alter their cell morphology as they assume a committed cell phenotype (Fig. 1a–c). In CGM, expansion media devoid of additional chemical inducers of differentiation, hASCs orient randomly in a multitude of directions as they grow. However, with the addition of mechanical stimulation in the form of 10% cyclic tensile strain (1 Hz, 4 hours/day) hASCs tend to align roughly perpendicular to the horizontal axis of strain (aligned between 0 ± 45° from the vertical axis) (Fig. 1d–f). This cellular orientation in response to strain was consistent independent of culture medium with all cell types demonstrating a proclivity to orient perpendicular to the axis of strain. Under static culture conditions, osteogenic hASCs show evidence of calcium accretion as visualized by accreted calcium crystals (denoted by blue arrows) over the cell monolayer surface (Fig. 1b). When undifferentiated and osteogenic hASCs are cultured under cyclic tensile strain, they exhibit an elongated morphology and appear highly oriented perpendicular to the axis of strain as compared to random orientation of the cells in the same induction media under static culture. Osteogenic hASCs exposed to strain also exhibit calcium accretion, however the crystal formations tended to be smaller, but more dispersed through the monolayer (Fig. 1e). Adipogenic hASCs tend to assume a more rounded cell phenotype indicative of adipogenic differentiation and show evidence of adipogenesis via accumulation of lipid vacuoles within the cells. Interestingly, in adipogenic cells, strain tended to encourage a slightly more elongated cell morphology and appeared to suppress formation of lipid vacuoles (Fig. 1c,f).

### Cyclic tensile strain upregulates osteogenic gene expression markers and downregulates adipogenic ones

**Figure 2:**
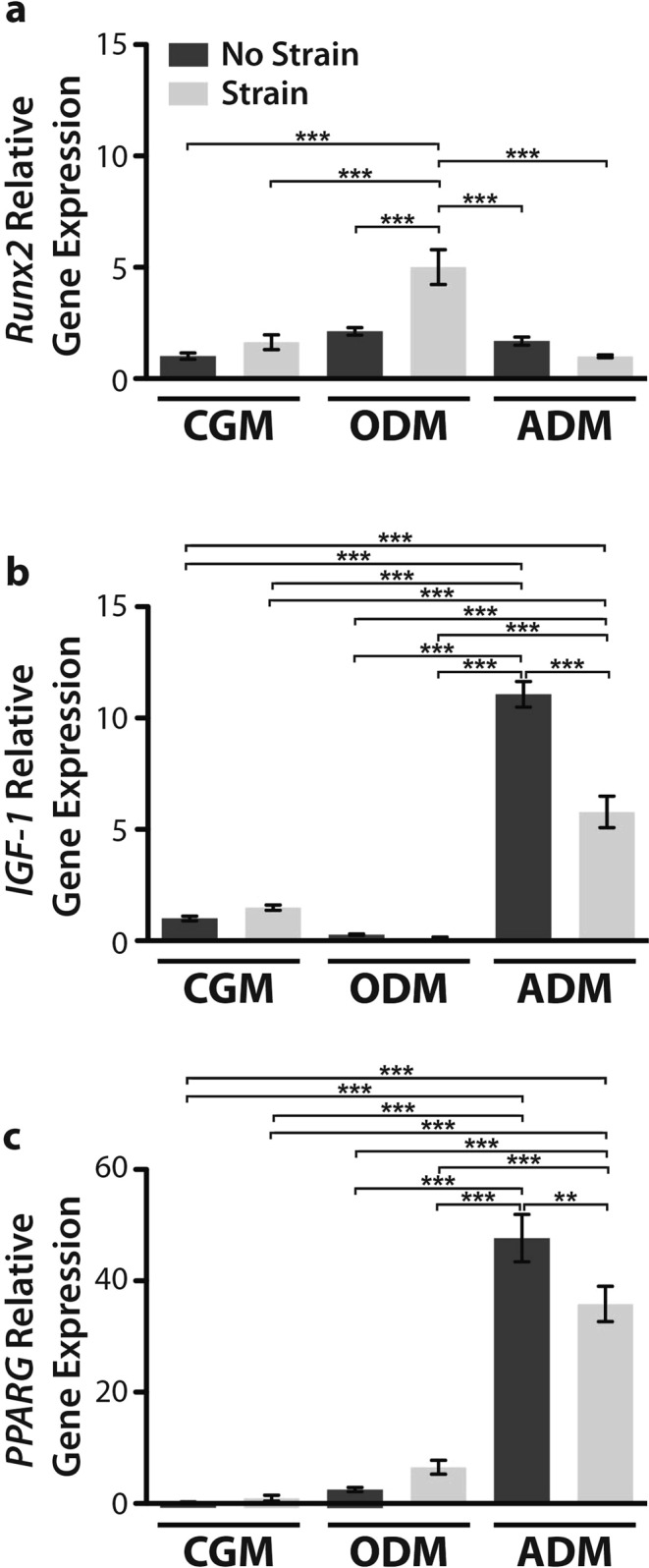
Gene expression of osteogenic *RUNX2* (**a**) and adipogenic gene expression markers *IGF-1* (**b**) and *PPARG* (**c**) following 3 days of culture. All cells were cultured under static (dark grey) and strained (light grey) conditions (strain at 10% cyclic tensile strain for 4 hours/day culture at 1 Hz). All values were normalized to hASCs cultured in complete growth medium without any induction media or mechanical stimulation. Cyclic tensile strain significantly upregulates baseline *RUNX2* expression levels in osteogenic hASC as compared to unstrained osteogenically differentiated cells (***p < 0.001). (**a**) Conversely, cyclic tensile strain significantly downregulates *IGF-1* (**p < 0.01) and *PPARG* gene expression in ADM samples. All gene expression data is reported as relative gene expression using the ddCT method. One-way ANOVA, Newman-Keuls multiple comparison post-hoc test, n = 3 independent cell samples. Error bars represent SEM.

In light of these general observations, we then examined early gene expression markers upregulated in osteogenesis, runt-related transcription factor-2 (*RUNX2*), and adipogenesis, insulin-like growth factor-1 (*IGF-1*) and peroxisome proliferator-activated receptor gamma (*PPAR-γ*), after just 3 days under inductive culture. In osteogenically differentiated hASCs, *RUNX2* expression was upregulated under exposure to cyclic tensile strain as compared to both undifferentiated hASCs and osteogenic hASCs under static culture (Fig. 2a). Under static culture with adipogenic induction medium, *IGF-1 and PPAR-γ* gene expression were both upregulated as compared to hASC cultured in expansion medium (Fig. 2b,c). Interestingly, exposure to cyclic tensile strain resulted in diminished adipogenic gene expression compared to hASCs under static culture, however, hASCs still exhibit a significant upregulation of *IGF-1 and PPAR-γ* in the presence of strain.

### Gene expression of cilia-associated proteins varies in a lineage-dependent fashion

**Figure 3:**
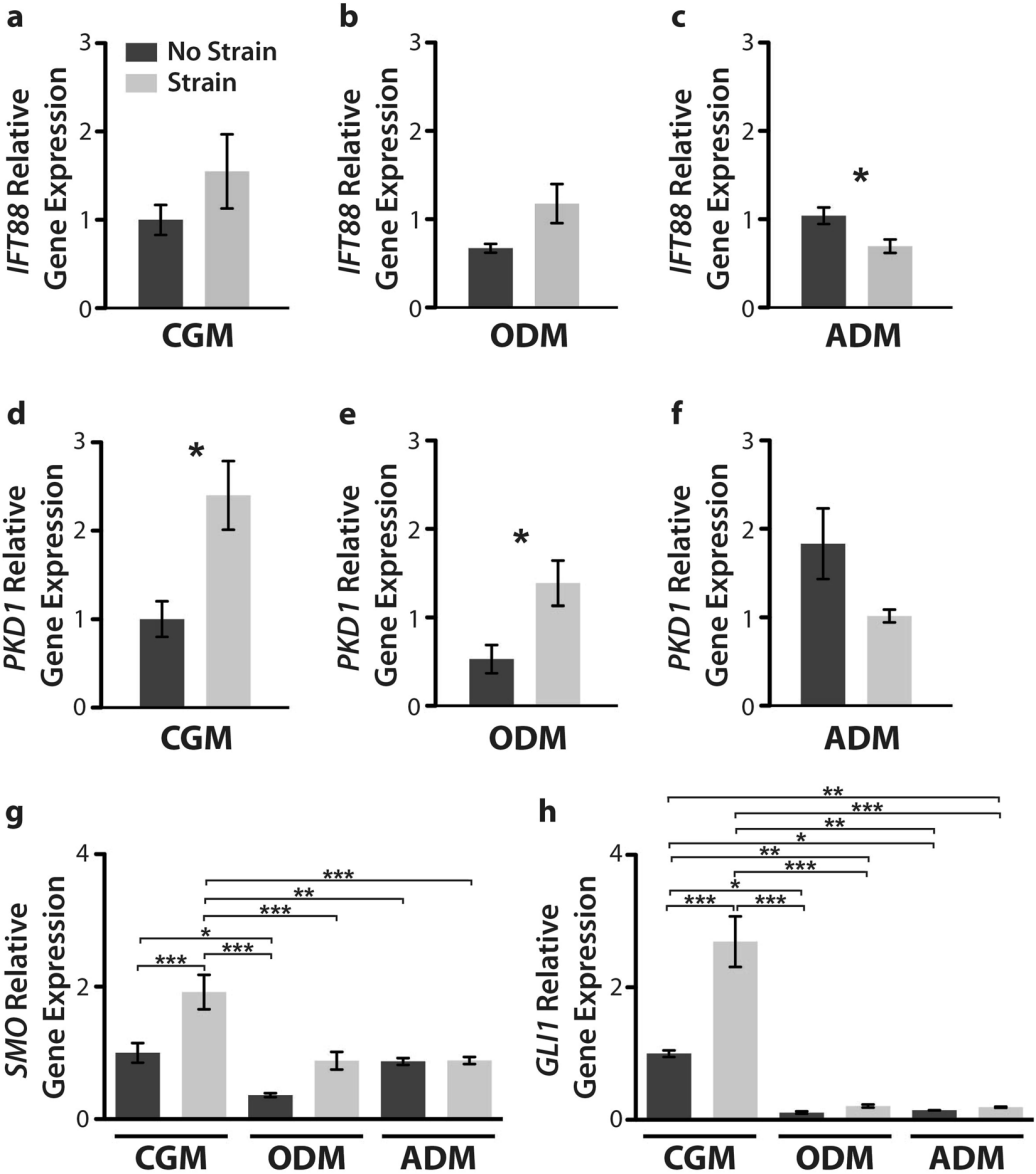
Gene expression of cilia-associated and Hedgehog signaling genes. Gene expression was analyzed following 3 days of culture in complete growth medium (CGM), osteogenic differentiation medium (ODM) or adipogenic differentiation medium under static (dark grey bars) or cyclic tensile strain (10% strain, 4 hours/day at 1 Hz) (light grey bars). Cyclic tensile strain moderately upregulates *IFT88* (**a**,**b**) and *PKD1* (**d**,**e**) gene expression in undifferentiated and osteogenically differentiated hASCs. Conversely, strain moderately downregulates *IFT88* (**c**) and *PKD1* (**f**) gene expression in adipogenically differentiated hASCs. Gene expression of hedgehog signaling genes *SMO* (**g**) and *GLI1* (**h**) exhibit higher expression in naïve hASCs as compared to osteogenically and adipogenically differentiated hASCs. Cyclic tensile strain upregulates both *SMO* (**g**) and *GLI1* (**h**) in naïve hASCs. All gene expression data is reported as relative gene expression using the ddCT method. For all genes n = 3, Error bars represent SEM. Student’s t-test (**a–f**) One-way ANOVA, Newman-Keuls multiple comparison post-hoc test (**g**,**h**) (*p < 0.05, **p < 0.01, ***p < 0.001).

To determine whether cilia structure and signaling dynamics may vary under exposure to cyclic tensile strain under differing types of chemical induction media, we analyzed the gene expression of cilia-associated proteins Intraflagellar transport protein 88 (*IFT88)* and polycystin 1 (*PKD1)* (Fig. 3a–f), known mediators of hASC osteogenesis^24^. We found that after 3 days of culture, *IFT88* was significantly downregulated in adipogenic hASCs exposed to strain (Fig. 3c) and *PKD1* gene expression was significantly upregulated in response to strain in both undifferentiated and osteogenically differentiated hASCs (Fig. 3d–e). To further investigate the functional expression of primary cilia-associated signaling molecules, we analyzed the gene expression of Hedgehog signaling genes smoothened (*SMO*) and glioma-associated oncogene-1 (*GLI1*). In undifferentiated hASCs, strain significantly upregulated *SMO* and *GLI1* expression as compared to hASCs in static culture (Fig. 3g,h). Further, GLI1 expression is significantly downregulated in osteogenic and adipogenic hASCs as compared to non-differentiated hASCs regardless of the mechanical culture condition (Fig. 3h). Osteogenic induction media significantly downregulated *SMO* gene expression compared the undifferentiated hASCs and this effect was seen in both the unstrained and strained conditions (Fig. 3g). Taken together, this suggests that primary cilia may exhibit a differential functional response to their surrounding mechanical environment depending on cell phenotype.

### Cilia length and morphology are distinctly different between osteogenic and adipogenic hASC cell populations

**Figure 4:**
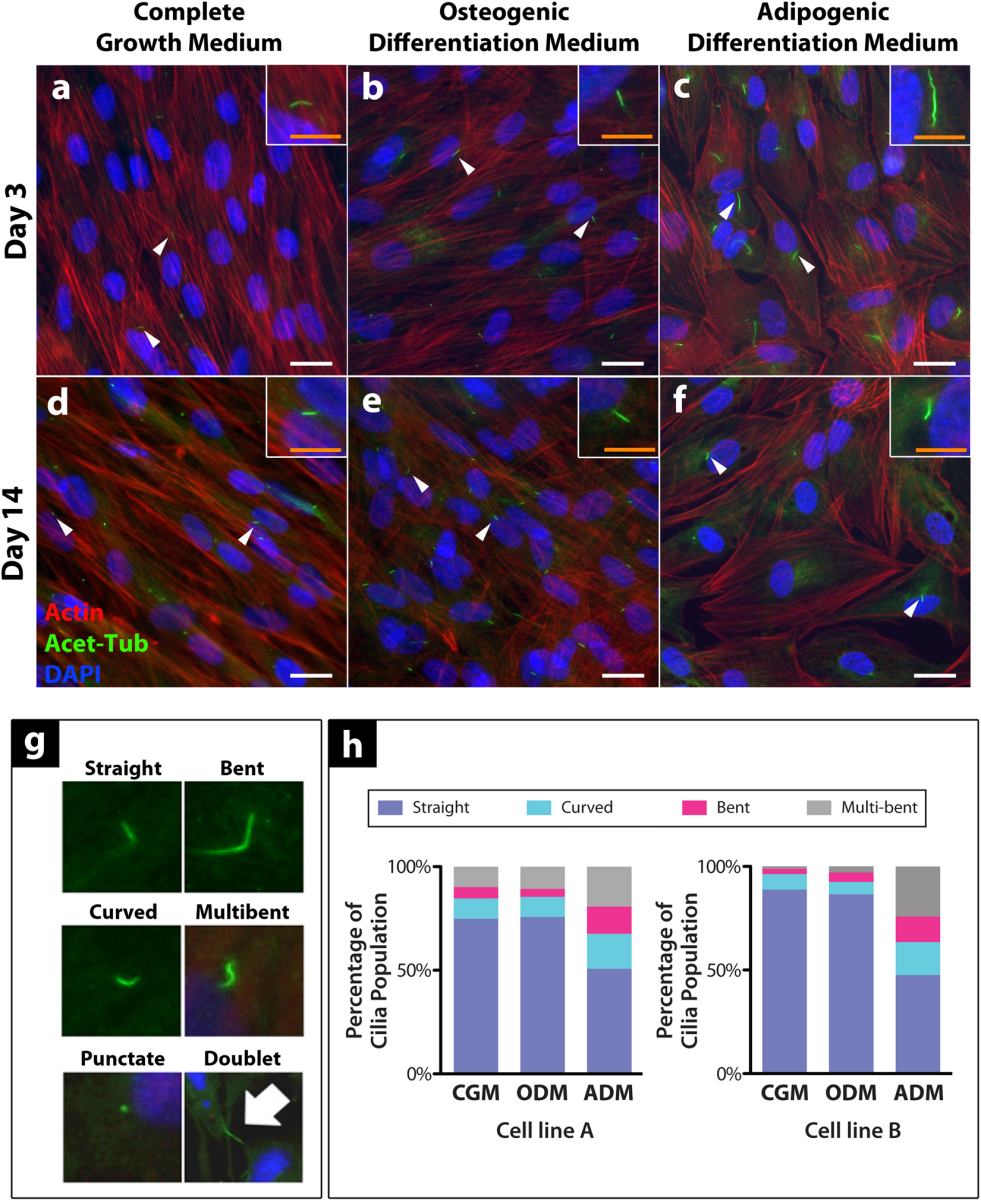
Primary cilia expression on differentiating hASC at day 3 (**a–c**) and day 14 (**d–f**) of culture. Primary cilia exhibit variation in their frequency of expression, length and conformation temporally throughout the differentiation process amongst different lineages. hASCs cultured in CGM (**a**,**d**) hASCs cultured in ODM (**b**,**e**) hASCs cultured in ADM. (**c**,**f**) White scale bar = 25 μm, Orange scale bar = 10 μm in zoomed image. Categorical analysis of primary cilia showing examples of cilia conformation frequently observed under immunofluorescence. (**g**) Doublets stained with acetylated tubulin are excluded from cilia frequency analysis as they are generally thought to be cytokinetic bundles, not cilia. Analysis of hASC primary cilia conformation following 3 days of culture under expansion medium (CGM), osteogenic differentiation medium (ODM) or adipogenic differentiation medium (ADM) for hASC lines derived from two separate donors (cell lines A and B). (**h**) Distribution of cilia conformation varies based on chemical stimulation and may be phenotype-specific. n > 750 cells analyzed for each media type.

To explore this concept further, we examined changes in cell morphology and primary cilia expression following 3 days and 14 days of culture under differing culture conditions (Fig. 4). We observed qualitatively that cell morphology changes in response to chemically-induced differentiation were concomitant with changes in cilia expression. Fewer cilia appeared to be expressed in hASC cultured in expansion media (Fig. 4a,d), however there was an increase in cilia expression with increasing osteogenic differentiation and assumption of an osteogenic cell morphology (Fig. 4b,e) (Table 1). Conversely, adipogenic cells appeared to prevalently express elongated cilia at day 3 of culture in ADM and a decreased number of cilia as they progressed towards a more committed adipogenic phenotype after 14 days of culture (Fig. 4c,f) (Tables 1 and 2).

**Table 1:** Prevalence of hASC primary cilia expression under cell growth (CGM), osteogenic differentiation (ODM) and adipogenic differentiation (ADM) medium at 3 days and 14 days in static culture.

		Day 3		Day 14	
		Average % Population Expressing Cilia	Standard Deviation	Average % Population Expressing Cilia	Standard Deviation
Cell Line A	Complete Growth Medium	34.08	±10.22	23.17	±4.33
	Osteogenic Differentiation Medium	19.51	±9.88	54.67	±9.78
	Adipogenic Differentiation Medium	44.14	±15.93	16.89	±5.05
Cell Line B	Complete Growth Medium	24.32	±9.30	56.58	±6.70
	Osteogenic Differentiation Medium	26.59	±15.43	61.06	±16.95
	Adipogenic Differentiation Medium	59.89	±14.38	38.30	±16.65

Differentiation confers a difference in the frequency of cilia expression in temporal and lineage-dependent fashion. n > 240 cells per media condition.

**Table 2:** A summary of apparent ciliary length as measured on immunofluorescent images of hASCs stained for acetylated α-tubulin.

Media	Average Cilia Length (μm)	Standard Deviation
Complete Growth Medium	1.43	±0.45
Osteogenic Differentiation Medium	1.38	±0.49
Adipogenic Differentiation Medium	3.14	±1.56

Cells were cultured on standard tissue culture-treated polystyrene plates for three days in cell growth (CGM), osteogenic differentiation (ODM) and adipogenic differentiation (ADM) medium. hASCs were grown to 85–100% confluency prior to the duration of the 72 hour media induction for the experiments. n > 300 cells per condition. derived from at least 3 biological replicates.

Following our qualitative analysis on primary cilia in hASCs cultured under different media conditions, we adapted a semi-quantitative categorical analysis approach to analyze changes in cilia conformation from Gardner *et al*.^20^ (Fig. 4g). We used this analysis to visualize the distribution of cilia conformations present in each culture condition at 3 days of culture under induction medium (Fig. 4h). In quantifying cilia conformation under expansion, adipogenic or osteogenic differentiation medium for three days, we found distinct differences in the conformations of cilia expressed on each hASC phenotype (Fig. 4h). Osteogenic and unspecified hASCs had comparable distributions of cilium shapes within their populations, while adipogenic hASC cilia expression was distinctly different (Fig. 4h). The data is presented here from two different donor cell lines to illustrate that this is phenomenon is not a donor line-specific effect. Interestingly, adipogenic medium resulted in the widest variation in the distribution of cilia shapes, however this may be due to the elongated cilium structure being more prone to bending. When considering the mechanosensitive properties of the primary cilium, it is important to consider the length and shape of the mechanosensor as that effectively changes the forces experienced by the structure.

### Mechanical stimulation modulates cell morphology and primary cilia length in a lineage-dependent manner

**Figure 5:**
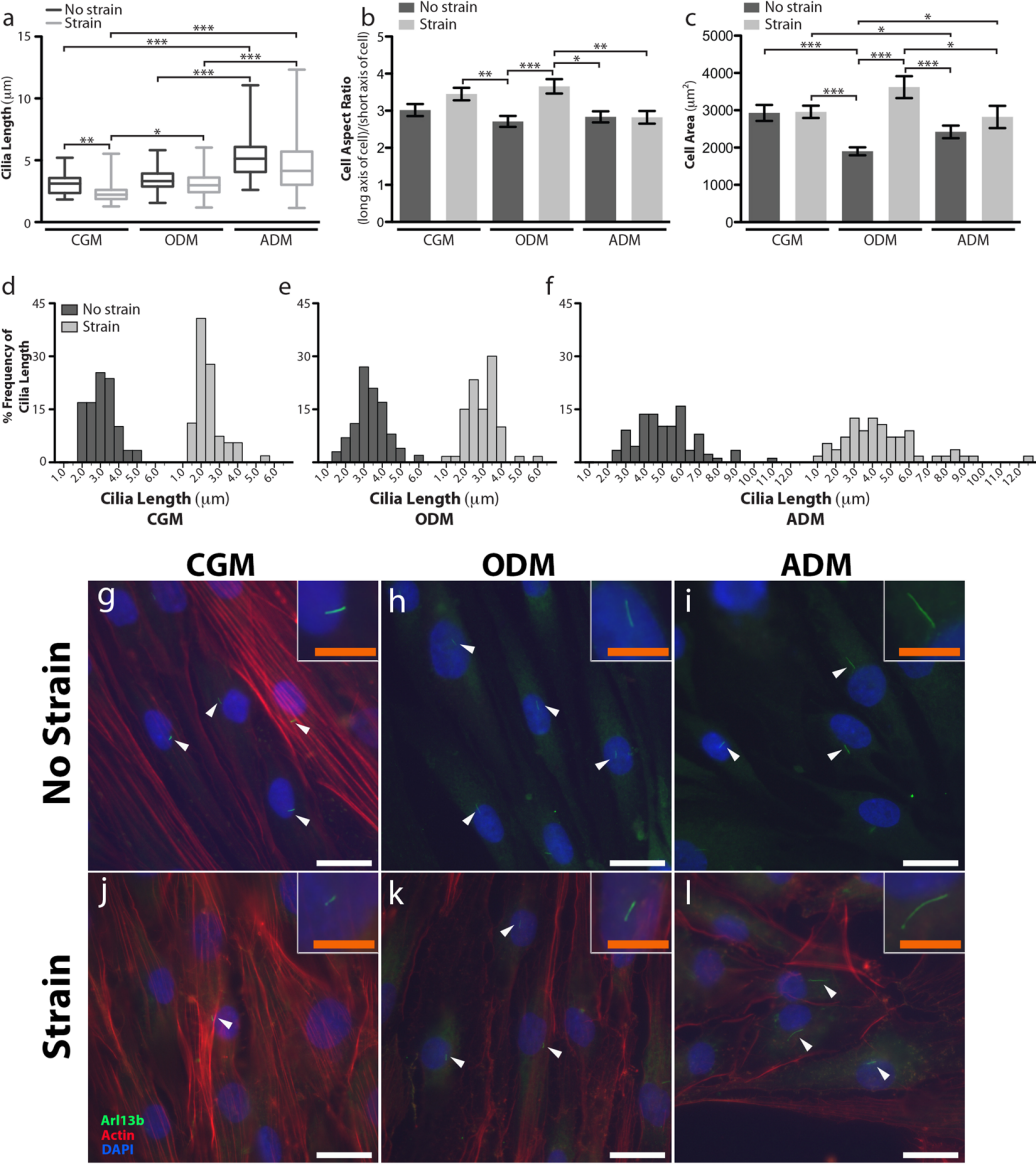
Semi-quantitative characterization of cilia and cell morphology under different chemical and mechanical culture conditions. (**a–f**) Average primary cilium length varies across culture conditions (**a**) also has a distinct lineage-dependent distribution of cilia lengths as seen by the histograms of cilia length in complete growth medium (CGM) (**d**) osteogenic differentiation medium (ODM) (**e**) and adipogenic differentiation medium (ADM). (**f**) Cell aspect ratio quantifies the length (long axis of the cell)/width (short axis of the cell) to assess cellular elongation (**b**) and cell area quantifies cell adhesion/spreading interaction with the culture substrate. (**c**) Both parameters were used to assess phenotype-specific morphology. n > 300 cells were characterized per culture condition. Analysis was performed following 3 days of culture under CGM (**g**,**j**), ODM (**h**,**k**) and ADM (**i**,**l**) on hASCs exposed to static culture (**g–i**) or mechanical stimulation in the form of 10% cyclic tensile strain (4 hours/day, 1 Hz) (**j–l**) on collagen I-coated silicone membrane BioFlex® plates. Cilia were identified with immunofluorescence using an antibody against ARL13b (green), actin (red) was stained with phalloidin and nuclei were stained with DAPI (blue). White scale bar = 25 μm, orange scale bar = 10 μm.

We next aimed to characterize the effect of mechanical stimulation on cilia length following culture under differentiation media and exposure to strain over the course of 3 days. We observed a general trend that cilia length seemed to decrease following exposure to 10% cyclic tensile strain in hASC, but this was only statistically significant in the complete growth medium condition (Fig. 5a,g,j). Generally speaking, primary cilia tended to be on average longer on hASCs under differentiation media, but this marked difference in average cilia length was most significant in adipogenically differentiated hASC in both the unstrained and strained conditions (Fig. 5a,g–l). Under strained conditions, osteogenically differentiated hASCs also expressed a longer mean cilia length as compared to undifferentiated hASCs cultured in CGM (Fig. 5a,j,k). Not only were cilia the longest in adipogenically differentiated cells, hASCs exhibit a wide distribution of lengths across the adipogenic cell population and strain appeared to enhance the wide variation in cilia length (Fig. 5d–f).

In addition to analyzing cilia length, we also semi-quantitatively characterized cell morphology. Interestingly, we found that cyclic tensile strain increased the aspect ratio of the cells cultured in CGM and ODM, but had no effect on adipogenic hASCs (Fig. 5b). Presumably this occurs concomitantly with cellular orientation roughly perpendicular to the axis of strain. We also analyzed cell area and strain seemed to only significantly affect this morphological parameter in osteogenically differentiating cells, with strain conferring an increase in cell area (Fig. 5c). A marginal increase in cell area was observed in adipogenically differentiated cells in response to strain, however this was not a statistically significant (Fig. 5c). Under static culture, it seems that the cell body of undifferentiated hASCs cover a larger area than differentiated cells, though the differentiated cells seem more sensitive to this morphological parameter as their cell area is increased in response to strain (Fig. 5c).

### Cyclic tensile strain enhances osteogenesis and suppresses adipogenesis

**Figure 6:**
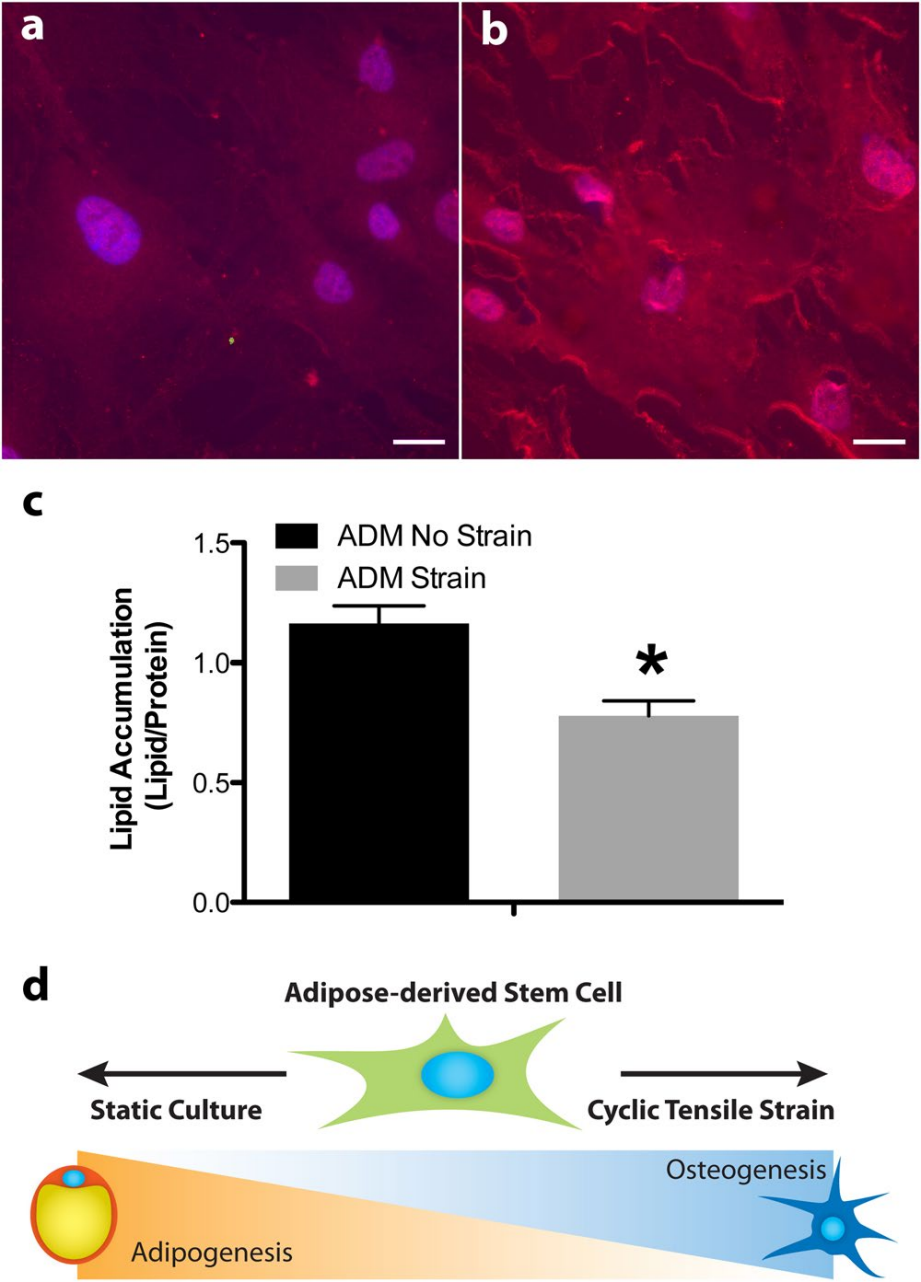
Osteogenic factors are upregulated under exposure to cyclic tensile strain and adipogenic factors are suppressed under exposure to strain. Runx2 nuclear expression in hASC in ODM (**a**,**b**) under static culture (**a**) and dynamic (10% cyclic tensile strain) culture (**b**) at 3 days. Lipid accumulation is suppressed in hASC cultured in adipogenic medium and cyclic tensile strain for 14 days. (**c**) Theoretic paradigm of mechanically-induced differentiation enhancing the osteogenic phenotype, while simultaneously suppressing the adipogenic phenotype. (**d**) Scale bar = 25 μm. Lipid quantification n = 3 independent monolayers, error bars represent SEM, *p < 0.05.

Our results show that both induction media and 10% cyclic tensile strain effects changes in cilia-associated gene expression, gene markers for adipogenic and osteogenic differentiation, as well as changes in cilia and cell morphology. Further, we observed qualitatively that nuclear RUNX2 protein expression was upregulated with culture in ODM under cyclic tensile strain (Fig. 6a,b). Concomitantly, adipogenesis is suppressed with exposure to 10% cyclic tensile strain following 14 days of culture in adipogenic medium as shown by diminished lipid accumulation (Fig. 6c).

## Discussion

Within mesenchymal and adipose-derived stem cell types, adipogenesis is generally thought to be at odds with osteogenesis with each lineage resulting in cell types of very different physiological function. This theoretical paradigm of stem cell differentiation (Fig. 6d) is in part supported by the signaling pathways involved in phenotypic differentiation, suggesting that adipogenic cell types are mutually exclusive with osteogenic cell types when undergoing these lineage commitment processes^33^. We hypothesized that 10% cyclic tensile strain may contribute to separating the two lineages further; that is to say that strain not only enhances osteogenesis of hASCs^7,12,16^, but may also suppress inherent adipogenic signals. *In vivo* and cellular studies in cell types other than hASC suggest that mechanical stimulation likely suppresses adipogenesis^34–36^, however it is unclear if a specific stimulation modality and/or magnitude optimally inhibits adipogenesis. Further, in this study we characterized primary cilium expression on hASCs and hASCs undergoing osteogenic or adipogenic lineage specification and highlight the potential differential function of this organelle in these different cell phenotypes.

Through our baseline analysis of hASC differentiation in CGM, ODM and ADM with and without strain, we observed stark differences between cell morphology and monolayer organization in hASC cultured in a static environment and hASC cultured under 10% cyclic tensile strain (1 Hz, 4 hours/day) (Fig. 1). There was evidence of osteogenic and adipogenic differentiation in both the static and strained cultures after 14 days in their respective induction media (Fig. 1). Irrespective of culture medium, the hASC tended to orient perpendicular to the axis of strain when exposed to strain, whereas non-mechanically stimulated cells exhibited a more random organizational pattern (Fig. 1). This is consistent with other studies on fibroblasts in response to cyclic tensile strain^37^, suggesting a realignment of the actin cytoskeleton perpendicular to the axis of strain prevents disruption of the internal tension within the cytoskeleton^38^. Interestingly, in the adipogenically differentiated cells, fewer cells appeared to accumulate lipid vacuoles than adipogenically differentiated hASC in static culture. This qualitative observation was the first sign that cyclic tensile strain may have an inhibitory effect on hASC adipogenesis in addition to our previous findings on hASC and hMSC osteogenesis^12,28,29^. Further, we observed qualitatively that though the adipogenic cells tended to align under strain, they maintained a more rounded morphology (Fig. 1f). This morphological observation was consistent with other studies of adipogenesis under strain, however the loading parameters and cell source differed from our study^39^.

To characterize the effect of 10% cyclic tensile strain on the differentiation of hASCs, we measured the expression of gene markers for osteogenesis (Fig. 2a) and adipogenesis (Fig. 2b,c) following 3 days of culture in induction media and daily exposure to strain. Cyclic tensile strain significantly upregulates *RUNX2* gene expression in osteogenically differentiated hASCs which supports our previously published observations of hASC calcium accretion upregulation under 14 days culture under strain (Fig. 2a)^12,40^. This upregulation of *RUNX2* gene expression has similarly been observed in MSCs exposed to mechanical stimulation in the form of oscillatory fluid shear^41^. IGF-1 is a secreted pro-adipogenic factor and PPARG is a nuclear receptor which is one of the central transcription factors in the adipogenic differentiation program^42^. Consistent with other adipogenic differentiation studies, *IGF-1* and *PPARG* gene expression were significantly upregulated under adipogenic differentiation, as compared to gene expression of hASCs cultured in CGM and ODM^43,44^. Interestingly, exposure to strain conferred a decrease in their expression (Fig. 2b,c). This suggests that mechanical stimulation in the form of strain quells the adipogenic gene expression program. However, under our loading parameters, the effect of mechanical stimulation is moderate compared to the effect of the chemical induction via adipogenic differentiation medium (Fig. 2b,c). Taken together our gene expression data is consistent with our qualitative observations of cell morphology following two weeks of culture, suggesting that strain not only enhances the osteogenic differentiation program, but suppresses the adipogenic one.

Our previous work in hASC strain-mediated osteogenesis^7,12,16^ and the role of the primary cilia in osteogenesis^24^, provided the impetus to explore the role of the primary cilium as a potential mechanosensor. Following daily exposure to 10% cyclic tensile strain and 3 days of stimulation in differentiation medium, we found that both *PKD1* and *IFT88* gene expression were upregulated under 10% cyclic tensile strain in undifferentiated hASC and osteogenically differentiated hASC (statistically significant for *PKD1* expression in CGM and ODM conditions) (Fig. 3d,e) and adipogenically differentiated hASC exhibited the opposite response (statistically significant for *IFT88* in ADM) (Fig. 3c). These inversely-related cilia gene expression findings suggested that the primary cilia may differentially sense the surrounding mechanical environment based on cell phenotype, in part validating their phenotype-specific function, particularly when considered with the osteogenic and adipogenic gene marker expression profiles with strain (Figs 2 and 3a–f).

To gain some insight into the how cilia-associated genes may be related to cilia function, we analyzed the gene expression of Hedgehog signaling molecules which localise to the primary cilium, *SMO* and *GLI1,* under each induction media both with and without strain (Fig. 3g,h). We found that strain significantly upregulated *SMO* and *GLI*1 in undifferentiated hASCs and likely activates hedgehog signaling, but this effect was not observed in osteogenic or adipogenic differentiated hASC (Fig. 3g,h). We found a marked downregulation of *SMO* and *GLI*1 gene expression with osteogenic and adipogenic differentiation. Our gene expression results were consistent with those reported by Plaisant *et al*. for MSCs undergoing osteogenesis suggesting that hedgehog signaling is decreased during osteogenesis^45^. Further, others have reported that hedgehog signaling inhibits adipogenesis, which supports our observed downregulation of *GLI1* in adipogenic hASCs^46,47^.

To further analyze the primary cilium structure in differentiated hASC, we used immunostaining to characterize the level of cilia expression and length of cilia elongation for each media type at 3 and 14 days of culture (Table 1 and Fig. 4). Our results illustrated stark differences in cilia expression amongst committed cell types, concomitant with changes in overall cell morphology over time in culture, particularly in the adipogenically differentiating cells (Fig. 4). We adapted a semi-quantitative categorical analysis approach from a report by Lavagnino *et al*. to analyze differences in cilia shapes among differing cell populations (Fig. 4g)^48^. In quantifying the cilia conformations observed on hASC cultured in expansion medium, adipogenic differentiation medium or osteogenic differentiation medium for 3 days, we found that each phenotypic population had a distinctly different distribution of cilia conformations (Fig. 4h). Adipogenically differentiated hASC had a stark change in the distribution of cilia shapes within the cell population, exhibiting a relatively higher percentage of cilia in curved or bent conformations (Fig. 4c,f,h). This is likely related to the increase in average length of cilia on adipogenically differentiated hASC (3.14 *μ*m ± 1.56) as compared to cilia observed in hASC cultured in CGM (1.43 *μ*m ± 0.45) or ODM (1.38 *μ*m ± 0.49) (Table 2), as the longer cilia may be more susceptible to bending. This may also be due to changing mechanical integrity of the cilia and a reduction in cilia rigidity, as proposed in an iPSC categorizing changes in cilia morphology with fibroblast reprogramming^49^.

Very few studies have explored changes in cilia expression with cell phenotype, however a study by Nathwani *et al*. reported observed changes in cilia length and presumed reductions in cilia rigidity in human induced pluripotent stem cells (iPSCs) derived from fibroblasts^49^, thus indicating that changes in cilia structure can change with phenotype. It is likely that the observed cilia morphologies in adipogenic hASCs may be due to both a change in cilia rigidity simultaneous with phenotype as well as observed cilia elongation making the cilium more susceptible to shape changes. A study on Bardet-Biedl syndrome proteins revealed that pre-adipocytes transiently express elongated primary cilia during the adipogenesis process, yet they lose their cilia upon terminal differentiation^50^. More recently, Dalbay *et al*. has reported that cilia elongation and recruitment of IGF-1Rβ were required for adipogenesis in cultured hMSCs^23^. The findings of these studies were consistent with our observations of cilia on hASC undergoing adipogenic differentiation, supporting the idea that ciliary elongation is a necessary step in adipogenic differentiation, but is likely independent of the hedgehog signaling (Figs 3g,h and 4c,f).

Extending this analysis further, we examined cilia expression on hASC cultured on the collagen I-coated silicone membranes of BioFlex® plates and compared the effects of 10% cyclic tensile strain on each hASC phenotype (Fig. 5). 10% cyclic tensile strain appeared to not only affect primary cilia conformation of each specified cell type, but also cell body morphology. Across all differentiation phenotypes, strain downregulated the average measured ciliary length, compared to the unstrained control, though this trend was only statistically significant in the hASCs cultured in CGM (Fig. 5a). This trend of decreasing primary cilia length under strain is consistent with observations in tendon explants and supports the idea of the “stress deprivation paradigm” stating that in the absence of mechanical stimulation, cilia lengthen, and in the presence of strain, the ciliary axoneme is shortened or resorbed^20^. This tendon work was one of the most direct demonstrations of the mechanically-induced length and shape changes of primary cilia in musculoskeletal tissues, however this response also been shown *in situ* on cartilage tissue^51^ and in cultured immortalized kidney cell lines^52^.

A further morphometric analysis on the hASCs cultured under each condition revealed interesting lineage specific trends. Strain significantly increased both the cell aspect ratio and cell spreading (cell area) of hASCs cultured in ODM (Fig. 5b,c). The hASCs cultured in CGM and ADM did not exhibit any statistically significant changes in these parameters, though CGM cells exhibited an increase in cell aspect ratio and ADM cells did seem to exhibit an increase in cell spreading (Fig. 5b,c,h,i,k,l). The adipogenic hASCs appeared to be particularly impervious to any strain-induced cell elongation. Taken together with the upregulation of RUNX2 expression in osteogenic hASCs cultured under strain (Fig. 6a,b) and the diminished lipid accumulation of adipogenic hASCs cultured under strain (Fig. 6c), these data suggests a relationship between strain-induced morphology changes and cell lineage commitment (Fig. 6d).

A study by Killian *et al*. showing that geometric cues could modulate osteogenesis and adipogenesis in hMSCs provides support for our findings. They reported that culturing hMSCs on rectangular or elongated geometries with a large aspect ratio enhanced osteogenesis and reduced adipogenesis^27^. These phenomena are largely thought to depend on myosin-generated cytoskeletal tension, with an increase in cytoskeletal tension leading to osteogenesis, while a decrease in cytoskeletal tension leading to adipogenesis and modulating cytoskeletal tension has been achieved through constraining cell shape^27,53^ and through tuning stiffness of the culture substrate^26^. It may be that the cyclic tensile strain applied to our osteogenic hASCs enhances already increasing cytoskeletal tension and as such results in enhances osteogenesis. However, within the limitations of our study, it is unclear whether the mechanical stimulation or the induced cell morphology changes which might be the driving factor in this process.

One of the primary aims of our study was to elucidate how the primary cilium may fit into the story of mechanotransduction and cell lineage specification. Based on the aforementioned principles of cell geometry and cytoskeletal tension, we can infer some basic information regarding the relationship between primary cilium length and lineage-specific cytoskeletal organization. Pitaval, *et al*. described a decrease in primary cilium length on RPE-1 cells (a retinal epithelium cell line) cultured on hard substrates compared to those cultured on soft substrates, indicating that the increased cytoskeletal tension on a stiff substrate resulted in shortened cilia length, whereas decreased cytoskeletal tension on a soft substrate resulted in elongated cilia^54^. We observed a similar trend with reduced cilia lengths on hASCs cultured on a hard substrate such as tissue culture plastic (Fig. 4 and Table 2) as compared to cilia lengths observed on hASCs cultured on the softer silicone membrane substrates (Fig. 5 and Table S1). Presumably, the elongated primary cilia may be a function of the reduction in cytoskeletal tension associated with the development of the adipogenic phenotype (Figs 4c,f, 5a,b,i,l and S1). Conversely, the increased cytoskeletal tension within the undifferentiated hASC and the development of an osteogenic phenotype results in the relatively shorter cilia (Figs 4b,e, 5a,b,g,h,j,k and S1). Furthermore, cyclic tensile strain is mechanically increasing cytoskeletal tension via stretch and as such results in the overall trend of a decreased average cilia length under strain (Fig. 5a). The large degree of variation observed in the cilia length data is likely due to a somewhat heterogeneous hASC population with cells starting at various levels of ‘stemness’ and/or cells differentiating towards their specified lineage at differing rates (Fig. 5a,d–f). In spite of this variation, it is clear that primary cilia are mechanosensitive and their expression corresponds to the specific cell phenotype and its expression is likely linked to cytoskeletal organization. Nevertheless, how these structures transduce signals to each other is still unclear.

Some have suggested that the primary cilium-mediated mechanotransduction functions through intracellular calcium signaling, the consensus mechanism by which the primary cilia in kidney epithelial cells transduce fluid flow. However, recent reports suggest that the mode of calcium signaling via the polycystin 1-polycystin 2 ion channel complex mechanism is still a point of debate^55,56^ and in the context of osteogenic cell types, the mechanism of cilia-mediated mechanotransduction seems to be different than in kidney epithelial cells. Malone *et al*. reported that cellular calcium flux under fluid flow is unaffected by disruption of the primary cilium in MC3T3-E1 osteoblasts, in contrast to the characteristic cilium-mediated calcium flux in MDCK kidney cells exposed to fluid flow^57^. A subsequent study using a FRET-based imaging approach in osteocytes demonstrated that fluid flow induces an increase in calcium within the ciliary compartment mediated by transient receptor potential vanilloid 4 (TRPV4), a mechanosensitive calcium channel which concentrates around the primary cilium^58^. More recently, similar TRPV4 functional activity in response to fluid oscillatory fluid flow has been implicated in the calcium flux of osteogenic murine MSCs^59^. We have reported that electrical stimulation-induced calcium flux in osteogenic hASCs on the cellular level is not mediated by the primary cilium per se^60^. However, the limited sensitivity of our experimental setup prevented us from resolving calcium flux in the ciliary microdomain. Though our study focuses on tensile strain, we suspect the potential mechanotransduction mechanisms in osteogenic hASCs maybe be somewhat analogous and clearly there is a vast opportunity to explore these mechanisms in future work.

This investigation provides some insight on the primary cilia expression during the lineage commitment process between osteogenic and adipogenic differentiation, but there is still a vast opportunity to understand how the biochemical and mechanical signaling activity of the primary cilium work together. The aforementioned “stress deprivation paradigm” in tendon is based on the principle that presumably a more extended cilium may detect smaller magnitude changes in the surrounding mechanical environment, and when larger magnitude mechanical forces are present, the cilium no longer requires such a large “lever arm” to sense mechanical signals^20^. This explanation follows for mechanically sensitive tissue such as bone, however these changes can also be explained through a cell physiology model, suggesting that strain confers changes in the ciliary signaling pathways such as hedgehog, and therefore the cell modulates cilia length to modulate hedgehog signaling activity^45^. In our study, undifferentiated hASCs exhibit a significant upregulation of *SMO* and *GLI1* gene expression in response to culture under cyclic tensile strain suggesting that exposure to strain specifically activates Hedgehog signaling in uncommitted hASCs (Fig. 3g,h). This increase in Hedgehog signaling is concomitant with a decrease in ciliary length in uncommitted hASCs, supporting that a physical change in the primary cilium, and thus in the Hedgehog signaling compartment, may play a direct role in modulating signaling activity (Fig. 5a). A recent study on MSC cultured on topographically grooved substrates expressed a higher average cilia length as compared to MSC cultured on flat substrates. The grooved substrates conferred reduced actin cytoskeletal organization, but this topography-induced cilia expression modification also modulated Wnt signaling^61^. It is likely that both models are accurate and there may be both a mechanical and biochemical component to how cilia respond to their mechanical surroundings in a lineage-dependent fashion.

It is possible that the mechanical rigidity of the cilium is changing along with phenotype as predicted by both chemical and mechanical induction and thus it is more susceptible to the bending influence of the tensile strain^49^. This likely occurs concomitantly with lineage-dependent changes in cytoskeletal and nucleoskeletal rigidity^62,63^. As the basal body of the primary cilium is docked at the cell membrane and its structure is formed by acetylated tubulin, the cilium is contiguous with the cytoskeleton; it follows that the rigidity of the primary cilium likely also changes with cytoskeletal tension.

Our results clearly indicated that both 10% cyclic tensile strain and lineage specific chemical induction media can modify cilia expression along with lineage changes. Evidence from this study and previous work in our lab established that 10% cyclic tensile strain enhances osteogenesis, further supported by upregulation of RUNX2 protein expression (Fig. 6a,b). Diminished lipid accumulation following 14 days of culture in adipogenic induction medium with exposure to strain further confirmed that osteogenesis is not only enhanced by 10% cyclic tensile strain, but also that adipogenesis is suppressed by strain (Fig. 6c). Suppression of adipogenesis in cell culture has been reported by others using immortalized mouse cell lines, however their strain parameters differed from our setup^39^.

When considering hASCs as a multipotent cell type, they have a general proclivity to become either cell phenotype, however adipogenesis and osteogenesis are considered to be orthogonal processes of differentiation and based on this theoretical principle^33^, it may be the case that 10% cyclic tensile strain diminishes baseline adipogenic signals as it enhances osteogenic signals. This is in line with work done in pre-adipocytes, illustrating the impact of exposure to a compressive force for 12 hours on diminishing adipogenic differentiation^36^. Our observations were also consistent with an *in vivo* mouse model study showing that MSCs in mice were less adipogenic when exposed to high frequency, low magnitude mechanical stimulation^34^. It is important to note a contrasting study which reported an enhancement of adipogenesis under exposure to strain, however the parameters of their strain system were quite different as their 3T3-L1 cells were exposed to static application of strain^64^. From our work, it is clear that 10% cyclic tensile strain reduces adipogenic differentiation, enhances osteogenic differentiation and these phenotypic changes also affect the primary cilium structure, a known mediator of cell differentiation.

In this body of work, we have demonstrated that hASC primary cilia exhibit a lineage-specific response to mechanical stimulation in the form of 10% cyclic tensile strain, however it is important to acknowledge the limitations of this study. Primary cilia are notoriously difficult to study under *in vitro* culture due to their variability in expression level, depending on cell cycle phase^65,66^. Further, their expression can additionally be modulated through cell plating density and serum starvation^66^, and their frequency of expression is not always predictable in a given stem cell population. hASCs are a somewhat heterogeneous cell population^67^ and the variation in cilia expression may be attributed to cell population subtypes, further complicating how the data is interpreted. Future work in this area may include enriching hASC population subtypes for a more in-depth analysis on primary cilia behavior.

Another caveat for consideration in this study is donor-to-donor variability in hASC^68–70^. In this study we used hASCs derived from three relatively age-matched female donors, known to exhibit an acceptable proclivity for both osteogenic and adipogenic differentiation. These donor hASC lines were already screened for multipotency and as such were preselected based on their ability to differentiate. Therefore, the results of this study are most relevant to hASC that exhibit similar characteristics. These results may have a limited relevance to hASC lacking the ability to osteogenically or adipogenically differentiate, however further research is required to better understand the physiological differences amongst hASC donor lines. We suspect that primary cilia expression patterns may be a potential biomarker indicative of the level of stemness of particular hASC sub-populations. Understanding their functional expression may help tease out some of the physiological differences between hASC donor lines, though this type of population sorting study was outside of the scope of this work.

It should be noted that this study provides limited data in regards to the mechanism by which cyclic tensile strain induces changes in hASC cilia-associated gene expression along with cilia length/morphology and how this corresponds to cilia function. Future work disrupting or modifying cilia expression under tensile strain and measuring calcium signaling and protein expression will be required for a full functional study to better understand how primary cilia on specific cell phenotypes sense their mechanical environment.

This is the first study to demonstrate that hASC primary cilia are mechanosensitive to uniaxial cyclic tensile strain and that hASC primary cilia exhibit phenotype-specific expression patterns during the hASC differentiation process. Further, we also showed that in hASCs osteogenesis is enhanced and adipogenic signals were quelled by exposure to 10% cyclic tensile strain. Taken together we have highlighted the importance of the primary cilium structure in mechanosensing and lineage specification and suggest that further work may identify this structure as a novel target in manipulating hASC for developing tissue engineering applications.

## Materials and Methods

### Cell isolation and expansion

Human adipose-derived stem cells (hASC) were obtained from waste adipose tissue derived from patients undergoing elective abdominal surgeries or liposuction procedures. Discarded tissue was collected in accordance to IRB exemption protocol #10-0201, under federal regulations [45 CFR 46.102 (d or f) and 21 CFR 56.102(c)(e)(l)]. This work was reviewed and all experimental protocols were approved by the Office of Human Research at UNC, and they determined that this study does not constitute human research as the cells are derived from discarded tissue samples, at University of North Carolina hospitals (Chapel Hill, NC). All data were analyzed anonymously and patient information obtained was limited to age, sex, ethnicity and site from which tissue was derived when available. Isolation of hASC was performed according to established protocols from our lab^6^, adapted from methods initially reported by Zuk *et al*. and Erickson *et al*.^2,71^. Once isolated, hASC obtained from approximately 50 g of tissue were allowed to propagate in culture in complete growth medium (CGM) until ~80% confluency (or up to two weeks). CGM contained Eagle’s Minimum Essential Medium, alpha-modified supplemented with 10% fetal bovine serum, 2 mM L-glutamine, 100 units/ml penicillin and 100 μg/ml streptomycin. The hASC were then trypsinized and frozen down at passage 0 (p0). All hASC used in these experiments were passage 4 or lower, and were derived from three female donors (ages 47–55 years). All three donor cells lines were validated for adipogenic and osteogenic differentiation and exhibited a similar proclivity to differentiate towards both lineages.

### Culture and differentiation under cyclic tensile strain

hASC were cultured in BioFlex® plates (Flexcell International, Hillsborough, NC) comprised of a culture surface composed of collagen I-coated silicone membranes. The hASC were cultured under both static and dynamic growth conditions. hASC cultured under mechanical stimulation were exposed to 10% cyclic tensile strain at 1 Hz, for 4 hours/day. In addition to the mechanical stimulation, they were cultured in three different media: complete growth medium (CGM), osteogenic differentiation medium (ODM) and adipogenic differentiation medium (ADM). ODM contained complete growth medium with the addition of 50 μM ascorbic acid, 0.1 μM dexamethasone, and 10 mM β -glycerolphosphate. ADM contained the aforementioned CGM supplemented with 1 μM dexamethasone, 5 μg/ml insulin, 100 μM indomethacin and 500 μM isobutylmethylxanthine. hASC were cultured in all media formulations both with and without tensile strain (Fig. 1).

### Visualization of hASC - phase contrast microscopy

hASC cultures were monitored using phase contrast microscopy throughout the duration of the culture/differentiation period for up to three weeks. Images were collected at day 17 of culture in the respective media types to record morphological changes in cell phenotype. Phase contrast microscopy allowed for detection of cell morphology, orientation, deposition of extracellular calcium crystals and formation of lipid vacuoles.

### RNA extraction and gene expression analysis

Quantitative RT-PCR analysis was used to determine relative mRNA expression levels of lineage specific gene markers for osteogenesis: Runt-related transcription factor 2, (Runx2) and adipogenesis: insulin-like growth factor-1 (*IGF-1*) peroxisome proliferator-activated receptor gamma (*PPARG*). Cilia-associated gene expression of *IFT-88*, *PKD1* and hedgehog signaling genes *SMO* and *GLI1* were used characterize potential ciliary activity under differentiation and strain. RNA cell lysate samples were collected following 72 hours in culture.

Lysates were run through a Qiashredder column (Qiagen, Valencia, CA) at 15,000 g for 2 minutes to homogenize samples. Total RNA was extracted using the RNeasy Mini Kit (Qiagen, Valencia, CA). RNA concentrations were measured using a NanoDrop spectrophotometer (Thermo Scientific, Wilmington, DE). To synthesize first strand cDNA, 110–600 ng of RNA in a 21 μL reverse transcriptase (RT) reaction was used with the Superscript III RT with oligo(dT) primers kit (Invitrogen, Carlsbad, CA). Taqman Gene expression assays with pre-determined primer-probe sets were used to amplify diluted (1:1) cDNA (Assays-on-Demand, Applied Biosystems, Foster City, CA). All gene expression profiles were normalized to hypoxanthine-guanine phosphoribosyltransferase (HPRT) (Assay Hs02800695_m1) in an ABI Prism 7000 system. Quantitative RT-PCR data was analyzed using the ddCT method of relative gene expression quantification and all gene expression data is reported as relative expression with n = 3 independent cell samples.

### Immunostaining

The hASC were fixed in 10% formalin for 15 minutes and rinsed three times in PBS. For strain experiments and static controls, the monolayers grown on the BioFlex® collagen I-coated silicone membranes were cut out of the plate for staining and visualization. For baseline cilia characterization and analysis under chemical stimulation, hASC were grown on coverslips. Following fixation, membranes or coverslips were transferred to a new plate and monolayers were blocked with a 0.2% Triton X-100/5.0% BSA stock solution for 40 minutes on a shaker table. Following blocking, the coverslips or membranes were incubated in the primary antibody solution in humidified chambers. Primary cilia were identified using mouse monoclonal antibodies against acetylated α-tubulin antibody (diluted 1:100) (Sigma, Catalog #T7451) or rabbit polyclonal antibodies against ARL13B (ProteinTech, Catalog #17711-1-AP) at a dilution of 1:50. The base of the cilium was identified with goat polyclonal antibodies against pericentrin (Abcam, Catalog #19044) at a dilution of 1:100, staining the centriolar base. Runx2 was used to identify osteogenic differentiation in response to strain and was visualized using a mouse polyclonal antibody against Runx2 at a dilution of 1:50 (Abcam). Staining for cell proliferation with ki67 was done using a mouse monoclonal antibody (Abcam, Catalog #ab8191) (Table S2) and staining for active β-catenin was done using a mouse monoclonal antibody (Millipore, Catalog #05-665) (Fig. S1). All antibodies were incubated in a solution of 0.2% Triton X-100, and 0.5% BSA. The cells were then incubated in the chambers overnight at 4 °C.

Samples were rinsed 3 times in PBS for five minutes each on a shaker table and then incubated in the secondary antibody (1:500), phalloidin 594 (Molecular Probes) (1:500) and DAPI (1:500) stain solutions. A chicken anti-mouse secondary was used against the mouse acetylated α-tubulin primary and goat anti-rabbit secondaries was used against the rabbit primaries. The samples were incubated for 3 hours at room temperature. Following the secondary incubation, the samples were placed in the final three rinses and PBS and excess liquid was blotted on a kimwipe in preparation for mounting. Prolong Gold Mounting Media (Molecular Probes, Eugene, OR) was used to mount the membranes and slides were allowed to dry in the dark for 24 hours prior to imaging.

### Categorical analysis of cilia conformation and cell morphology

Semi-quantitative image analysis with ImageJ was used to measure variations in frequency of cilia expression, cilia conformation, cilia length and orientation, cell aspect ratio and cell orientation in static and actively strained hASC in 2D culture. Additionally, we adapted a method described by Gardner *et al*. to categorically analyze cilia conformation to parse out changes in cilia structure following culture in various differentiation media (Fig. 4g)^20^. For each media condition (CGM, ODM, ADM) under static culture at 3 and 12 days of culture, n > 750 cells (cell count identified by DAPI staining), across n = 3 independent cell monolayers (Fig. 4). For cells exposed to cyclic tensile strain, n = 3 biologically independent cell monolayers were cultured on BioFlex® plate collagen I-coated silicone membranes and n > 300 cells were analyzed across these samples per culture condition (Fig. 5).

### Adipogenesis analysis

Lipid accumulation was measured to detect evidence of adipogenic differentiation. A colorimetric adipogenesis absorbance assay (Biovision, Milpitas, CA) was used to quantify triglyceride content. Following culture in adipogenic differentiation media for 14 days under static culture or under cyclic 10% strain at 1 Hz, 4 hours/day, cells were scraped in 200 μL lipid extraction buffer (Biovision, Milpitas, CA), collected into microfuge tubes and incubated at 90–100 °C for 30 minutes on a heat block, and then stored at −30 °C. Lysate was then thawed, spun down at 1000 rcf for 2 minutes and the supernatant was transferred to a new tube to eliminate debris interference with absorbance readings. The sample was then diluted 1:1 with adipogenesis assay buffer (Biovision, Milpitas, CA) and 50 μL of each lysate was prepared according to the manufacturer’s protocol. Briefly, the 50 μL sample was combined with 46 μL adipogenesis assay buffer, 2 μL Adipogenesis Probe (Biovision, Milpitas, CA), and 2 μL Adipogenesis Enzyme Mixture (Biovision, Milpitas, CA) and absorbance was read using a 96 well plate on a Tecan Microplate Reader (Tecan Group Ltd, Switzerland) using Magellan^TM^ Data Analysis Software (Tecan Group Ltd, Switzerland) at a wavelength of 570 nm. Adipogenesis measurements were normalized to protein content using a colorimetric bicinchoninic acid (BCA) absorbance assay (Thermo Scientific Pierce, Rockford, IL). Remaining adipogenesis samples were processed according to the manufacturer’s protocol for the BCA assay and samples were read at a wavelength of 560 nm.

### Statistical analysis

A Student’s t-test with 95% confidence interval was applied to all data analyzing two conditions. All data with three or more conditions were analyzed using a one-way ANOVA with a Neuman-Keuls multiple comparison post-hoc test. For gene expression and end-product expression data, n = 3 independent biological replicates, with p-values denoted as follows: *p < 0.05, **p < 0.01, ***p < 0.001.

## Supplementary information

Supplementary Data and Legends
