# Supplementary material for: Primary Cilia Exhibit Mechanosensitivity to Cyclic Tensile Strain and Lineage-Dependent Expression in Adipose-Derived Stem Cells
Source: Sci Rep. 2019 May 29;9:8009. doi: 10.1038/s41598-019-43351-y (PMC6541635; doi:10.1038/s41598-019-43351-y)
Supplement: Supplementary file 1 — Supplementary Data and Legends [file 41598_2019_43351_MOESM1_ESM.pdf]

Primary Cilia Exhibit Mechanosensitivity to Cyclic Tensile Strain and Lineage-Dependent Expression in Adipose-Derived Stem Cells

Josephine C. Bodle[1], Mehdi S. Hamouda[1], Shaobo Cai[2], Ramey B. Williams[1], Susan H. Bernacki[1], Elizabeth G. Loboa[1, 3]

[1]Joint Department of Biomedical Engineering, University of North Carolina at Chapel Hill and North Carolina State University, Raleigh, North Carolina, 27695; [2]Department of Materials Science and Engineering, North Carolina State University, Raleigh North Carolina, 27695; [3]College of Engineering at University of Missouri, W1051 Thomas & Nell Lafferre Hall, Columbia, MO 65211

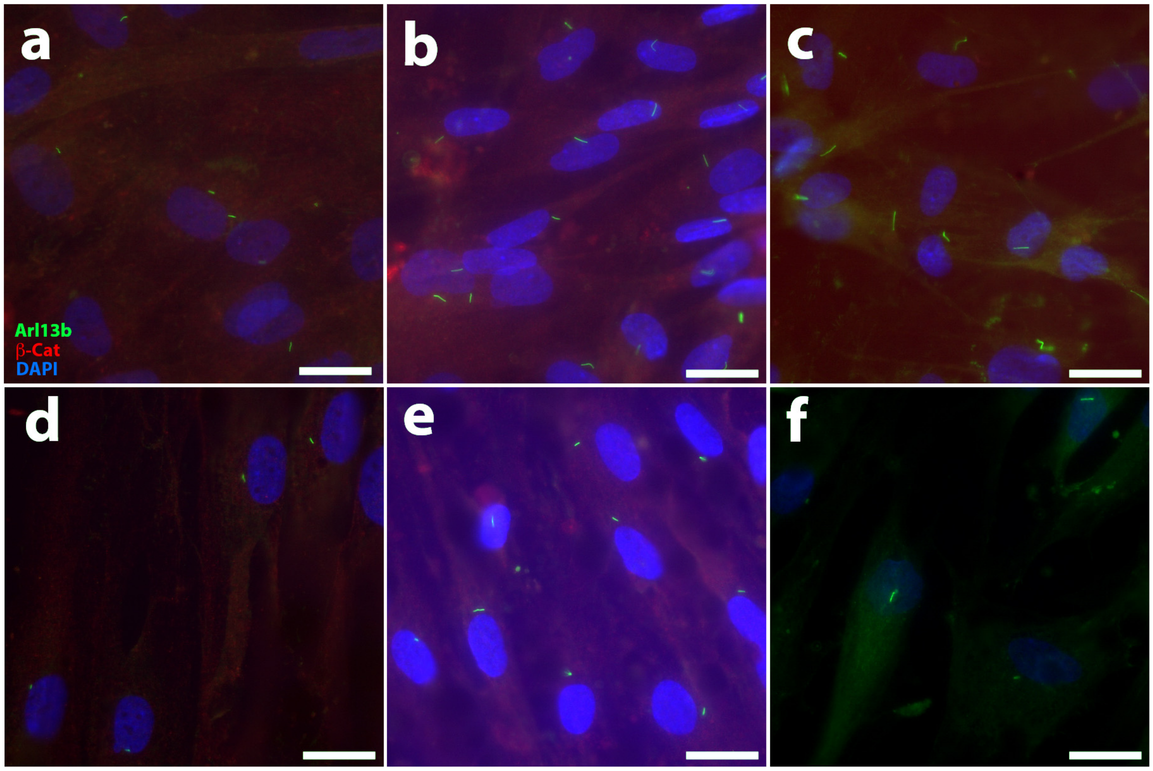

Supplemental Figure S1. Active β-catenin expression in hASCs under various culture conditions. hASCs were cultured in complete growth medium (CGM) (a, d), osteogenic differentiation medium (ODM) (b, e) and adipogenic differentiation medium (ADM) (c, f) in the absence (a-c) or presence of 10% cyclic tensile strain (1Hz, 4 hours/day) (d-f) for 17 days. Immunofluorescent staining was used to identify active β-catenin (red), cilia Arl13b (green) and DAPI was used to stain nuclei (blue). Cytoplasmic active β-catenin expression is observed in undifferentiated and osteogenically-differentiated hASCs, but it is not seen in adipogenically-differentiated hASC, both in the presence and absence of strain. Qualitatively, active β-catenin expression appears upregulated in osteogenic hASCs, though more comprehensive expression analysis is necessary to confirm this qualitative finding. Scale bar = 25 μm.

**Media**	**Static Culture Average Cilia Length (µm)**	**Standard Deviation**	**Cyclically Strained Culture Average Cilia Length (µm)**	**Standard Deviation**
Complete Growth Medium	3.09	±0.78	2.38	±0.77
Osteogenic Differentiation Medium	3.39	±0.87	3.01	±0.83
Adipogenic Differentiation Medium	5.18	±1.60	4.57	±2.20

Table S1. A summary of apparent ciliary length as measured on immunofluorescent images of hASCs cultured on collagen I-coated silicone membranes of Flexcell Bioflex plates. hASCs were cultured on membranes to 85-100% confluency in complete growth medium (CGM) and subsequently cultured in specific induction medium CGM, osteogenic differentiation medium (ODM) and adipogenic differentiation medium (ADM) for 72 hours. Strained hASCs were exposed to 10% cyclic for 4 hours/day at 1Hz. n>300 cells per condition.

	ki67+ Expression				Primary Cilia Expression			
	Static		Strained		Static		Strained	
**Media**	**Percentage ki67+ hASCs**	**Standard Deviation**	**Percentage ki67+ hASCs**	**Standard Deviation**	**Percentage Cells Expressing Primary Cilia**	**Standard Deviation**	**Percentage Cells Expressing Primary Cilia**	**Standard Deviation**
Complete Growth Medium	37.6%	±22.1%	32.9%	±15.6%	19.9%	±7.2%	22.6%	±11.5%
Osteogenic Differentiation Medium	57.6%	±10.3%	44.7%	±21.0%	31.3%	±9.7%	27.9%	±13.3%
Adipogenic Differentiation Medium	30.7%	±8.9%	17.0%	±17.4%	51.5%	±5.9%	38.6%	±10.8%

Table S2. Quantification of actively proliferating and primary cilia expression on subconfluently cultured hASCs exposed to various culture media and mechanical stimulation (static culture in the absence of strain and 10 % cyclic tensile strain for 4 hours/day at 1Hz). hASCs were cultured on collagen I-coated silicone membranes of Flexcell Bioflex plates in complete growth medium (CGM) for 24 hours. At 24 hours, media was changed to fresh CGM, osteogenic differentiation medium (ODM) or adipogenic differentiation medium (ADM) for the subsequent 52 hours. Cyclic tensile strain was applied 3 times over the culture period—the first time following the media change at 24 hours, and 2 subsequent times at 24 hour intervals for the duration of the experiment. Membranes were fixed following the final application of strain (approximately 52 hours following induction media and initial application of strain). n>500 cells per condition.
